# Integration of Genotoxic Biomarkers in Environmental Biomonitoring Analysis Using a Multi-Biomarker Approach in Three-Spined Stickleback (*Gasterosteus aculeatus* Linnaeus, 1758)

**DOI:** 10.3390/toxics10030101

**Published:** 2022-02-22

**Authors:** Amélie Cant, Marc Bonnard, Jean-Marc Porcher, Jean Prygiel, Audrey Catteau, Laurence Delahaut, Olivier Palluel, Cyril Turiès, Alain Geffard, Anne Bado-Nilles

**Affiliations:** 1 Institut National de l’Environnement Industriel et des Risques (INERIS), UMR-I 02 SEBIO, Parc Technologique Alata, BP 2, 60550 Verneuil-en-Halatte, France; jean-marc.porcher@ineris.fr (J.-M.P.); audrey.catteau@univ-reims.fr (A.C.); olivier.palluel@ineris.fr (O.P.); cyril.turies@ineris.fr (C.T.); 2 Université de Reims Champagne-Ardenne (URCA), UMR-I 02 SEBIO, UFR Sciences Exactes et Naturelles, Campus Moulin de la Housse, BP 1039, 51687 Reims, France; marc.bonnard@univ-reims.fr (M.B.); laurence.delahaut@univ-reims.fr (L.D.); alain.geffard@univ-reims.fr (A.G.); 3 Agence de l’Eau Artois-Picardie, 200 Rue Marceline—Centre Tertiaire de l’Arsenal, BP 80818, 59508 Douai, France; jean.prygiel@orange.fr

**Keywords:** biomonitoring, biomarkers, three-spined stickleback, genotoxicity

## Abstract

Water is impacted by a variety of increasing pressures, such as contaminants, including genotoxic pollutants. The proposed multi-biomarker approach at a sub-individual level gives a complementary indicator to the chemical and ecological parameters of the Water Framework Directive (WFD, 2000/60/EC). By integrating biomarkers of genotoxicity and erythrocyte necrosis in the sentinel fish species the three-spined stickleback (*Gasterosteus aculeatus*) through active biomonitoring of six stations of the Artois-Picardie watershed, north France, our work aimed to improve the already existing biomarker approach. Even if fish in all stations had high levels of DNA strand breaks, the multivariate analysis (PCA), followed by hierarchical agglomerative clustering (HAC), improved discrimination among stations by detecting an increase of nuclear DNA content variation (*Etaing*, *St Rémy du Nord*, *Artres* and *Biache-St-Vaast*) and erythrocyte necrosis (*Etaing*, *St Rémy du Nord*). The present work highlighted that the integration of these biomarkers of genotoxicity in a multi-biomarker approach is appropriate to expand physiological parameters which allow the targeting of new potential effects of contaminants.

## 1. Introduction

In order to protect, restore and use sustainable water resources of continental hydrosystems, the European Commission set up the Water Framework Directive (WFD, 2000/60/EC) which aims at achieving good water quality in 2027 [1] by promoting the chemical and ecological status of freshwaters. Nevertheless, the chemical approach ignores toxicity, as well as mixture effects of contaminants [2], and ecological assessment is often considered as an a posteriori approach lacking preliminary information on toxicity [3], despite the inclusion of biomarkers in the WFD revision being promoted by scientists [2,4,5,6]. What is more, some biomarkers are already used in the descriptor 8 (D8C2) of the Marine Strategy Framework Directive (MSFD, 2008/56/EC) to assess the biological effects of contaminants [7,8].

Monitoring biomarkers at the sub-individual level is a way of gaining more knowledge about early-warning signals of potential long-term alterations to population, community and ecosystem health. Therefore, multi-biomarker approaches are increasingly recommended to reflect the main physiological states of an organism [9,10,11]. Notably, this approach is relevant to evaluating the ecotoxicity of surface waters, which are regarded as receptacles of a huge diversity of xenobiotics [12].

Among teleost fish used for continental hydrosystem biomonitoring, the three-spined stickleback (*Gasterosteus aculeatus*) presents various advantages for ecotoxicology studies. This species is a major component of shallow-water food webs in the northern hemisphere. It is tolerant to temperature and salinity variation, is small and easy to handle [13], allowing its caging in large geographical areas [11]. Sticklebacks are also relatively tolerant to pollution [9,14]. In addition, multi-biomarker approaches have been already developed for this sentinel species, including biomarkers of innate immune responses, neurotoxicity, digestive enzyme activities, metabolic detoxification and the antioxidant system [9]. Different integration tools, such as clustering and IBR, are used to synthetize and express the results obtained for these different biomarkers [11,15,16,17]. These biomarkers have proven their relevance for active biomonitoring [15,18,19]. The optimal conditions of stickleback caging have already been characterized to minimize the impact on biomarker responses [19,20,21]. However, the monitoring of genotoxicity at different scales in the frame of active methods has not been included in these multi-biomarker approaches in stickleback, although these effects are important to consider.

Indeed, a large number of pollutants can have genotoxic effects [22], i.e., impacts directly or indirectly affecting the integrity of a cell’s genetic material (DNA, RNA), which can lead to mutagenic, carcinogenic or teratogenic effects [23]. Among biomarkers, genotoxic effect is considered as a major endpoint to assess aquatic pollution-related toxicity [24,25] affecting organisms at all trophic levels [24,26,27]. Different degrees of DNA damage can be distinguished, i.e., primary DNA damage (potentially reparable), mutations and chromosomal damage (clastogenic and/or aneugenic effects, unrepairable) [28]. One of the most promising biomarkers to assess genotoxicity in aquatic species for biomonitoring is to measure DNA strand breaks by a comet assay [24]—a sensitive, reliable and fairly inexpensive method [29]. The alkaline version of the comet assay (single cell gel electrophoresis assay, SCGE) allows the detection of alkali labile sites, DNA double- and single-strand breaks (ALS, DSBs and SSBs) [30] and requires a small number of cells [31]. This tool has been largely used with peripheral blood erythrocytes of fish to assess the impact of environmental chemicals in biomonitoring studies [32,33,34,35,36]. Indeed, non-mammalian vertebrates, such as teleost fish, still have nucleated erythrocytes with low nuclease activity [37], which allows the measurement of the accumulation of DNA damage. This cellular model is easy to obtain and gives a high density of erythrocytes already dissociated. This is an advantage for carrying out multiple assays on the same sample. For all these reasons, fish erythrocytes are interesting and widely used for the assessment of genotoxicity [33]. At the chromosomal scale, flow cytometry (FCM) may be applied to assess variations in the nuclear DNA content of a large population of cells, including micronuclei, which are defined as irreversible events [38]. The DNA content variation can be assessed by the adaptation of the protocol developed for peripheral blood cells of fish [39]. FCM has already demonstrated its relevance to detecting chromosomal damages in blood cells of fish exposed to various conditions [40,41,42].

**Figure 1:**
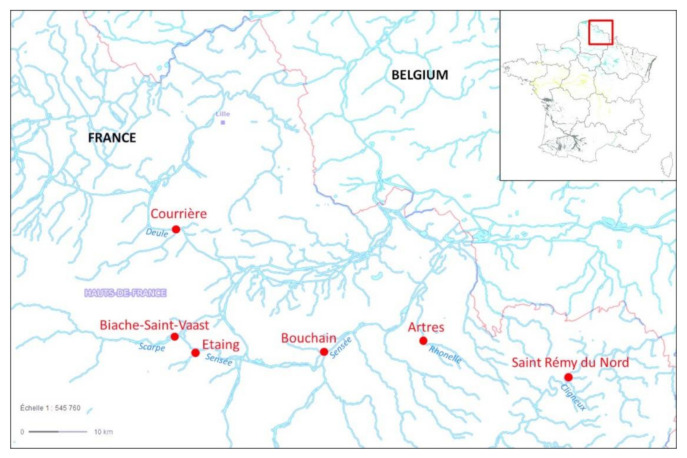
Location of all sampling stations investigated in the Artois-Picardie watershed, north of France.

Many xenobiotics can impact DNA integrity at different scales, but until today few studies have assessed jointly DNA strand breaks, chromosomal damage and erythrocyte mortality in fish, especially in stickleback. Monitoring at different scales allows an integrated view of the genotoxic risk for aquatic biomonitoring, as recommended by Orsière [28]. The first objective of this work was to gain more knowledge of the responses of biomarkers of genotoxicity and erythrocyte mortality, which are assessed by the comet assay and flow cytometry methods. Therefore, the innovative point was to assess genotoxicity at different scales of the genome on peripherical erythrocytes of the three-spined stickleback. The second objective was to determine whether these biomarkers could improve the existing stickleback multi-biomarker approach and contribute to the discrimination of different stations. More broadly, the aim was to make an ecotoxicological diagnosis of the water quality of six stations located on the Artois-Picardie watershed, north France (Figure 1). Sampled stations were recommended by the Water Agency for the inconsistency between their chemical and ecological status based on the WFD parameters.

## 2. Materials and Methods

Each experiment was conducted in accordance with the European directive 2010/63/UE on the protection of animals used for scientific purposes at INERIS facilities (registration number E60-769-02). For the present work, all fish came from a well-characterized population of the French National Institute for Industrial Environment and Risks (INERIS) (Verneuil-en-Halatte, Oise, France) which were maintained in an outdoor pool with natural vegetation and macro-invertebrate communities.

### 2.1. Experimental Design and Sampling Sites

In Autumn 2019, 30 sticklebacks, with a balanced sex ratio and homogeneous length (47.04 ± 3.76 mm) and weight (1.47 ± 0.4 g) were caged in a 36 L cylindrical structure in six distinct stations in the north of France (Figure 1, Table 1). The Cligneux river at the *Saint-Rémy du Nord* station (Nord, France) and the Rhonelle river at the *Artres* site (Nord, France) were considered as polluted small and natural streams [35,43], poorly urbanised and characterised by a strong pressure of agricultural practices. The Scarpe at the *Biache-Saint-Vaast* station (Pas-de-Calais, France) was considered highly polluted [35,43] and was located on an artificial canal. The Deule at *Courrières* (Pas-de-Calais, France) was a highly modified river used as a waterway. The Sensée river was studied on two points near *Bouchain*, (*Sensée Bouchain*, Nord, France), where the river was natural, and at the *Etaing* station (*Sensée Etaing*, Pas-de-Calais, France), a small stream surrounded by agricultural practices. All these sites were contaminated by polycyclic aromatic hydrocarbons (PAHs) [44]. Temperature, dissolved oxygen, pH and conductivity were monitored at the first day and after 21 days of caging for each station (Table 1).

After 21 days of caging, as recommended [21] and already tested by Catteau et al. [11,15,45], sticklebacks were directly anesthetised in situ by balneation with MS222 (tricaine methanesulfonate, 100 mg/L, Tricaine Pharmaq, Overhalla, Norway) to avoid handling stress and then killed by cervical dislocation. Each fish was weighed, measured and sexed and each organ was sampled for further analysis. Briefly, 2 µL of blood was collected and directly diluted in 120 µL of citrate buffer [39]. Then, 50 µL of blood was maintained at 4 °C ± 1 °C until erythrocyte mortality analysis; the rest was stored at –80 °C for genotoxicity analysis. Liver and muscle tissues were collected, weighed and stored at −80 °C for biochemical analysis (approximately 10 mg for muscle). Spleens were removed, pressed through 40 µm sterilized nylon mesh with 1 mL Leibovitz 15 medium modified with penicillin and streptomycin (both at 500 mg/L) and maintained at 4 °C for 12 h before immune analyses to eliminate any bias due to stressful conditions of fish sacrifice [46].

### 2.2. Morphometric Indices

Fulton’s condition index (K) was calculated as (total body weight/standard length^3^) × 100 [47]. The hepatosomatic index (HSI) [48] and the gonado-somatic index (GSI) [49] were calculated as (100 × (organ weight/total body weight)).

### 2.3. Biomarkers Analysis

#### 2.3.1. Peripherical Erythrocyte Counts, Mortality and Genotoxic Biomarkers

The 50 µL of blood maintained at 4 °C was diluted with 150 µL of citrate buffer before performance of the flow cytometry analysis (MacsquantX, Miltenyi Biotec, Bergisch Gladbach, Germany). The peripherical erythrocyte counts were detected using forward scatter (FSC—size of cells) and side scatter (SSC—complexity of cells) parameters. Erythrocyte mortality was detected using a double labelling method with YO-PRO^®^-1 (1 mM in DMSO, Invitrogen^TM^ Thermo Fisher Scientific, Bothell, WA, USA) for apoptotic cells (B1, green fluorescence) and propidium iodide (1 mg/mL in water, Invitrogen^TM^ Thermo Fisher Scientific, Bothell WA, USA) for necrotic cells (B3, red fluorescence) as preconized for leucocytes by Bado Nilles et al. [46]. After 10 min of incubation on ice and in darkness, samples were analysed after cell excitation by a 488 nm argon laser.

Chromosomal damage was determined for erythrocytes according to the method developed by Vindeløv & Christensen [39] and adapted for turbot by Goanvec et al. [50] and Marchand et al for stickleback [41]. Briefly, erythrocytes were adjusted, under 4 × 10^7^ cells/mL, before successive treatments to disrupt plasma membranes to allow access to DNA, disrupt RNA and stabilise amino acids and mark DNA. A sample of stabilized chicken red blood cells (Fitzgerald) was used as a standard and analysed at the same time as the fish blood samples. Each FL3 coefficient of variation (CV) corresponds to a measure of nucleotide size. DNA damage corresponds to the CV of fish erythrocyte samples minus the CV of chicken red blood cell samples.

The primary DNA strand break level was assessed by the comet assay on cryopreserved erythrocytes according to the protocol defined by Singh et al. [30] and previously adapted for three-spined sticklebacks by Santos et al. [35,51] with some modifications. All analyses were realized at 4 °C with inactinic light (dim red light) to avoid DNA damage. The blood samples were rapidly defrosted (18 s) in a 37 °C-bath, one-fiftieth (1/50) diluted with PBS buffer to adjust cell density and finally half-diluted with agarose LMP (low melting point, type VII, 1% *w*/*v* in PBS, 0.01 M, pH 7.4, without Ca or Mg) and maintained in liquid state at 37 °C. After chilling (15 min on ice), the slides were treated in several baths to disrupt plasma membranes to access nucleoids (lysing solution, Ph 10, 1 h) to obtain single-stranded DNA (denaturing buffer, Ph > 13, 40 min) and DNA strand breaks (SSB, DSB and ALS) (electrophoresis, 24 min, 20 V, 500 mA, 6 W). Then, the slides were washed (neutralization buffer, twice 20 min), dried (absolute ethanol, 10 min) and kept at room temperature and in the dark until their staining with Sybr Green 1 X, according to the supplier’s recommendations. Thereafter, slides were scanned with a fluorescence microscope (EVOS^TM^ FL Auto 2, Invitrogen^TM^ Thermo Fisher Scientific, Bothell WA, USA, optical filter for Sybr Green (497 nm/520 nm)). For each slide, 200 comets by fish (corresponding to 100 nucleoids/gel with two gels for one slide for each fish) were scored by an image analysis system (Comet Assay IV version 4.3.1, Instem, Stafforshire, UK). To express DNA strand breaks, the percentage of tail intensity (TI%), corresponding to the percentage of damaged DNA, was chosen, according to Langie et al. [52]. For each gel (100 comets) the median of tail intensity was calculated. Comet assay results for the fish were expressed as the means of the two gel medians.

#### 2.3.2. Innate Immune Capacities and Biochemical Parameters

Concerning immunomarkers, splenic leucocyte isolation was performed following the protocols described previously by Bado-Nilles et al. [53]. Then, analyses were carried out of whole leucocytes, using a MacsquantX flow cytometer (Miltenyi Biotec, Bergisch Gladbach, Germany). For each leucocyte sample, 10,000 cells were counted. Granulocyte–macrophage percentages, cellular mortality (apoptotic and necrotic leucocytes), leucocyte respiratory burst [46], lysosomal [53] and phagocytosis activity, corresponding to capacity and efficacity [54], were measured.

Concerning the antioxidant system and metabolic detoxification, livers were homogenized and centrifuged. The resulting post-mitochondrial fraction was used for biomarker measurements. Protein concentrations were determined using the method of Bradford [55]. Hepatic activities of lipidic lipoperoxidation (TBARS), superoxide dismutase (SOD), total glutathion (GSH), glutathion peroxydase (GPx) and catalase activity (CAT) were assessed according to the method developed by Ohkawa et al., Paoletti et al., Vandeputte et al., and Paglia & Valentine respectively [56,57,58,59,60]. Ethoxyresorufin-O-deethylase (EROD) and glutathione-S-transferase (GST) activities, involved in metabolic detoxication, were analysed following Flammarion et al. and Habig et al. [61,62]. All these biochemical assays were adapted for sticklebacks by Sanchez et al. [9,43,63]. Synaptic transmission was assessed by the cholinesterase activity (ChE) measured in S9 muscle fractions following the method of Ellman [64].

### 2.4. Statistical Analysis

All statistical analyses were performed with R software version 3.6.1. Before any other statistical tests, normality was checked using Shapiro’s tests (*p* ≤ 0.05) and homogeneity of variance was tested using Levene’s test (*p* ≤ 0.05). When the normality assumption was not fulfilled, data were log-transformed if this improved normality. Then, to assess the difference between stations in function of sex, each biomarker was analysed separately via a two-way ANOVA (package *car*) followed by a Tukey test (package *lsmeans*) for parametric data or two Kruskal–Wallis tests (package *stats*) on site and sex factors separately, followed by a Nemenyi test (package *agricolae*) for non-parametric data. Finally, hierarchical agglomerative clustering (HAC), including a standardised principal component analysis (PCA) (package *FactoMineR*), was performed. The purpose of these tests was to explore the relationship between biomarkers and the stations’ characteristics and to illustrate the discrimination of stations (package *FactoMineR*). Information about the biomarkers contributing to the build of each cluster and the percentage of fish from each site gathered in the same cluster were collected. All hypotheses were tested at the level of *p* ≤ 0.05. The statistical analyses are detailed in the Tables S2–S4.

## 3. Results

The number of fish collected after 21 days of exposure and the responses of all biomarkers for each group of sticklebacks in each station are shown in Table 2 (Mean ± SD). These raw values were used for all the analyses presented below.

Twenty-nine fish were recovered in *Bouchain* and *Etaing*, 28 in *Rémy du Nord*, *Artres*, *Biache-Saint-Vaast* and only 11 in *Courrières*. The sex ratio was near 50/50 for each site. For all the fish studied, out of 153 sticklebacks collected, 51.6% were males and 48.4% were females. A slight mortality was observed, with one or two fish per cage for all sites, except for fish caged in *Courrières*, where a huge individual mortality (63%) was recorded.

Globally, based on measured biomarkers, two types of stations would appear. *Courrières* and *Bouchain* seemed to be characterized by low responses of innate immune capacities but with a high respiratory burst index and high cholinesterase activity. The four other stations (*St Rémy du Nord*, *Artres*, *Biache-Saint-Vaast* and *Etaing)* would be affected by high immune capacities, high antioxidant system responses and genotoxicity. With the aim of improving understanding of the results, a data analysis through a principal component analysis (PCA) followed by hierarchical agglomerative clustering (HAC) was proposed.

### 3.1. Effect of Current Biomarkers on Site Discrimination

**Figure 2:**
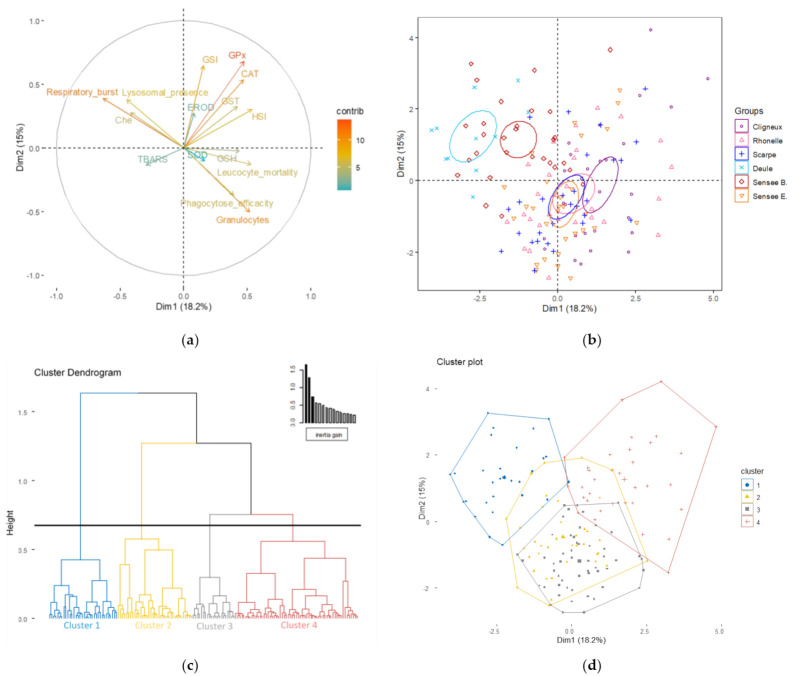
Results of the principal component analysis (PCA) with biometric, biochemical and immune biomarkers (first and second component) followed by the hierarchical agglomerative clustering (HAC). PCA: (**a**) variables graph and (**b**) individual graph showing 95% confidence ellipses around the barycenter of different groups. Each dot represents one fish. (**c**) HAC represented by a dendrogram and (**d**) by an individual graphical cluster.

The repartition of stations as functions of biometric, biochemical and immune responses was explained by 33.2% of the overall variance in the first two principal components of the PCA (Figure 2, Table S1). The first axis represented 18.2% of variance because of the respiratory burst index (14.80% of contribution), HSI (10.54% of contribution) and leucocyte mortality (10.29% of contribution). The second component explained 15.0% of variance, mainly GPx (20.51% of contribution), GSI (18.54% of contribution), CAT (12.72% of contribution) and granulocyte–macrophage percentages (11.28% of contribution) (Figure 2, Table S1).

The fish caging in *Courrières* and *Bouchain* formed globally one group in the PCA, which was mostly divided by the first dissimilarity node separating Cluster 1 from the others around 1.6 on the HAC, constituted of 100% of fish from *Courrières* and 58.6% from *Bouchain* (Table 3, Figure 2).

As shown with the PCA and the HAC (Figure 2, Table 4), the specificity of the fish caged in these two stations concerned their high respiratory burst index (6.95 ± 2.38 U for *Courrières*, 3.87 ± 1.94 U for *Bouchain*, Table 2; v-test of 9.9 for Cluster 1, Table 4) and ChE activity (65.34 ± 24.12 U/g protein for *Courrières*, 43.03 ± 21.66 U/g protein for *Bouchain*, Table 2; v-test of 5.8 for Cluster 1, Table 4), their weak granulocyte–macrophage percentages (40.36 ± 7.42% for *Courrières*, 39.52 ± 9.39% for *Bouchain*, Table 2; v-test of −5.6 for Cluster 1, Table 4) and their lower phagocytosis efficiency (10.95 ± 2.22% for *Courrières*, for 13.01 ± 4.09% *Bouchain*, Table 2; v-test of −5.3 for Cluster 1, Table 4). Nevertheless, *Bouchain* was also under-represented by the other clusters (13.8%, 20.7% and 6.9% of fish from Clusters 2, 3 and 4 respectively, Table 3).

Another group was also defined with the same PCA corresponding to fish from *St Rémy du Nord*, *Artres*, *Biache-St Vaast* and *Etaing* (Figure 2). More details for these stations were obtained with the HAC (Figure 2, Table 3 and Table 4). Indeed, the fish from *St Rémy du Nord* were spread between Cluster 3 (53.6% of fish) and Cluster 4 (46.4% of fish), which were represented by a lower condition index of reproductive system for males and a higher level for females concerning GSI (0.88 ± 0.31 for males and 2.36 ± 0.54 for females, Table 2; v-test of −6.4 and of 6.1 for Cluster 3 and 4 respectively, Table 4), higher HSI (3.55 ± 0.67 for males and 4.27 ± 1.08 for females, Table 2; v-test of 5.7 for Cluster 4, Table 4), higher granulocyte–macrophage percentages (53.47 ± 7.46%, Table 2; v-test of 5.3 for Cluster 3, Table 4), higher GPx activity (17.49 ± 4.76 for males and 50.22 ± 25.77 for females, Table 2; v-test of 6.8 for Cluster 4, Table 4) and higher catalase activity (4.59 ± 2.42 U/g prot × 10^3^ for males and 7.84 ± 2.52 U/g prot × 10^3^ for females, Table 2; v-test of 7.2 for Cluster 4, Table 4). The three other stations (*Artres*, *Biache-St Vaast* and *Etaing*) were represented by Cluster 2 (7.1% of fish from *Artres*, 67.8% of fish from *Biache-St* *Vaast*, 44.8% of fish from *Etaing*) in addition to Clusters 3 (57.1% of fish from *Artres*, 17.8% of fish from *Biache-St Vaast* and 37.9% of fish from *Etaing*) and 4 (37.7% of fish from *Artres*, 14.3% of fish from *Biache-St Vaast*, 17.2% of fish from *Etaing*) (Table 3). Cluster 2 concerned higher phagocytosis efficiency (19.08 ± 6.81% for *Artres*, 25.76 ± 6.70% for *Biache-St Vaast*, 18.65 ± 7.26% for *Etaing*, Table 2; v-test of 5.8, Table 4), higher SOD activity (3.06 ± 0.66 U/g prot × 10^3^ for *Artres*, 2.82 ± 1.09 U/g prot × 10^3^ for *Biache-St Vaast* and 3.42 ± 0.56 U/g prot × 10^3^ for *Etaing*, Table 2; v-test of 5.1, Table 4) and higher TBARS content (130.14 32.40 nmol/g prot for males, 123.58 ± 48.95 nmol/g prot for females for *Artres*, 153.09 ± 33.30 nmol/g prot for males, 143.19 ± 23.78 nmol/g prot for females for *Biache-St Vaast* and 153.37 ± 56.04 nmol/g prot for males, 143 ± 37.68 nmol/g prot for females for *Etaing*, Table 2; v-test of 5.8, Table 4).

### 3.2. Evolution of Site Discrimination by Adding Genotoxic Biomarkers

**Figure 3:**
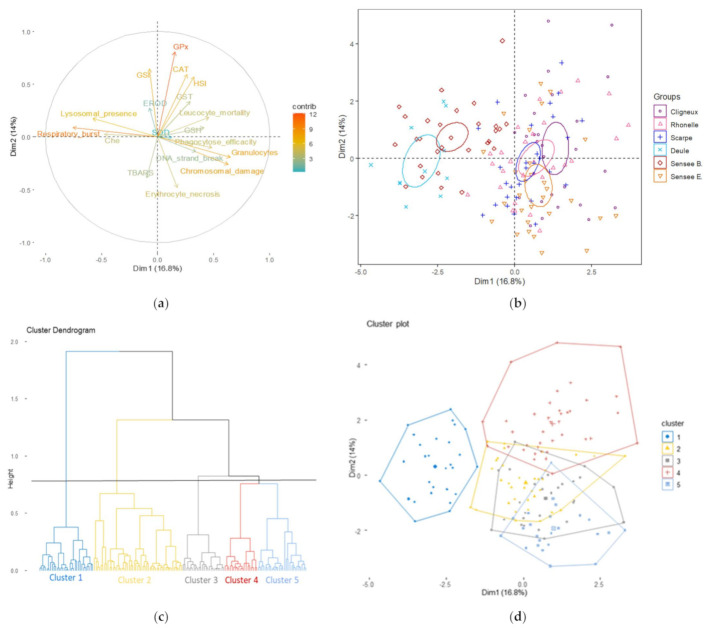
Results of the principal component analysis (PCA) with biometric, biochemical, immune and genotoxic biomarkers integrated in the battery (first and second component) followed by the hierarchical agglomerative clustering (HAC). PCA: (**a**) variables graph and (**b**) individual graph showing 95% confidence ellipses around the barycenter of different groups. Each dot represents one fish. (**c**) HAC represented by a dendrogram and (**d**) by an individual graphical cluster.

The inclusion of genotoxic biomarkers into the analysis improved site discrimination mainly along the first axis of the PCA (Table 3 and Table 4, Figure 3) and made a good contribution for chromosomal damage (13.06% of contribution) on the first axis and for erythrocytes necrosis (9.12% of contribution) on the second axis, inducing a little less of the overall variance of the PCA (30.8%). The first axis, which represented 16.8% of variance, was still mainly characterised by respiratory burst index (18.90% of contribution) and by some other immunomarkers that were also prevalent, such as granulocyte–macrophage percentages (13.90% of contribution) and lysosomal presence (11.00% of contribution). The second axis, which explained 14.0% of variance, was still built by GPx (25.67% of contribution), GSI (16.47% of contribution) and CAT (13.91% of contribution) and now by HSI (12.72% of contribution) (Figure 3, Table S1).

As shown in the first analysis, caged fish in *Courrières* and *Bouchain* formed globally one group better separated in this second PCA by higher correlation with the first axis (Figure 3, Table S1) and by the first dissimilarity node separating Cluster 1 from the others around 1.3 in the HAC (Figure 3). Always, 100% of the fish from *Courrières* and now 62.1% from *Bouchain* (Table 3) fell completely under Cluster 1, as demonstrated by the HAC. This group was still characterised by a higher respiratory burst index (v-test of 9.8, Table 4), higher neurotoxicity (v-test of 5.9, Table 4), weak phagocytosis efficiency (v-test of −5.1, Table 4) and low granulocyte–macrophage percentages (v-test of −5.5, Table 4) and was modified for the most part by the better representation of lysosomal presence (334.09 ± 52.36% for males, 378 ± 66.64% for females for *Courrières*, and 409.31 ± 90.56% for males, 462.36 ± 78.41% for females for *Bouchain* Table 2, v-test of 5.4, Table 4) and by the addition of negative CVs of DNA content (-0.60 ± 2.18 for *Courrières* and −2.34 ± 1.35 for *Bouchain*, Table 2, with v-test of −5.9, Table 4). *Bouchain* was still also reflected by three other clusters (24.1%, 3.5% and 10.3% for Clusters 2, 3 and 4 respectively, Table 3).

Another group was still constituted by this second PCA (Figure 3) corresponding to the fish caged in the same sites (*St Rémy du Nord*, *Artres*, *Biache-St Vaast* and *Etaing*) already highlighted by the first PCA (Figure 2). A HAC, more detailed due to the formation of a fifth cluster, was obtained with the inclusion of genotoxic biomarkers (Figure 3, Table 4). Most of the fish caged in *Etaing* (51.7% of fish), some of those at *St Rémy du Nord* (25% of fish) and only 3.6% of the fish of *Artres* and *Biache-St-Vaast* were gathered in Cluster 5 (Table 3). Cluster 5 was mainly characterised by higher erythrocyte necrosis only at *Etaing* (11.99 ± 7.09% for males and 6.11 ± 3.58% for females) and *St Rémy du Nord* (9.21 ± 7.50% for males and 1.56 ± 0.67% for females, Table 2; v-test of 8.6, Table 4) and by higher positive CVs of DNA content (3.87 ± 4.72 CV for *Etaing* and 2.41 ± 2.35 CV for *St Rémy du Nord*, 2.41 ± 2.19 CV for *Artres* and 2.31 ± 2.78 CV for *Biache-St Vaast* Table 2, v-test of 5.4, Table 4). Cluster 2 was only characterised by higher SOD activity (v-test of 5.1, Table 4), and Cluster 4, which corresponded to higher GPx activity (v-test of 7.8, Table 4), higher CAT activity (v-test of 7.2, Table 4), higher GSI (v-test of 6.5, Table 4) and higher HSI (v-test of 5.6, Table 4) characterised *Etaing* (31.0% and 17.2% of fish, respectively, Table 3), *St Rémy du Nord* (7.1% and 46.7% of fish, respectively, Table 3), *Artres* (25.0% and 32.1% of fish, respectively, Table 3) and *Biache-St-Vaast* (32.1% and 28.6% of fish, respectively, Table 3). Cluster 3 concerned only *St Rémy du Nord* (21.4% of fish, Table 2), *Artres* (39.3% of fish, Table 2) and *Biache-St-Vaast* (35.7% of fish, Table 3) and is represented especially by higher leucocyte mortality (10.46 ± 3.77% for leucocyte necrosis and 8.70 ± 3.75% for leucocyte apoptosis for *St Rémy du Nord*, 12.33 ± 3.54% for leucocyte necrosis and 7.14 ± 3.30% for leucocyte apoptosis for *Artres*, 10.29 ± 4.24% and 5.27 ± 2.53% for *Biache-St-Vaast*, Table 2; v-test of 5.7, Table 4). HSI, GSI and SOD activities are well represented in this clustering, but statistical analysis showed non-significant differences between stations for these three parameters (*p* > 0.05) (Table 2).

Furthermore, even if the biomarker of DNA strand breaks measured by the comet assay was not well projected in the PCA obtained, this parameter made a lower but significant contribution to Clusters 2 and 3 (v-test of -3.6 and 4.6 respectively, Table 4). The majority of fish from *St Rémy du Nord* (21.4%, Table 3), from *Artres* (39.3%, Table 3) and from *Biache-St-Vaast* (35.7%, Table 3) were found in Cluster 3, characterised by a higher level of DNA strand breaks (28.74 ± 16.92% TI for *St Rémy du Nord*, 38.78 ± 19.69% TI for *Artres*, 25.28 ± 13.69% TI for *Biache-St-Vaast*, Table 4), whereas *Etaing* (31.0% of fish, Table 3) and *Bouchain* (24.1% of fish, Table 3) were located especially in Cluster 2, characterised by the lowest DNA strand breaks levels (22.36 ± 12.70% TI for *Etaing* and 18.94 ± 7.85% TI for *Bouchain*, Table 4).

## 4. Discussion

Many xenobiotics can impact DNA integrity, but until today few studies have assessed jointly DNA strand breaks, chromosomal damage and erythrocyte mortality in three-spined sticklebacks. The innovative aspect of this work was to assess genotoxicity at different scales of the genome in peripherical erythrocytes of this sentinel species in the context of active biomonitoring. The first objective was to gain more knowledge of biomarker responses of genotoxicity and erythrocyte mortality. The second was to determine whether these biomarkers could improve the already existing multi-biomarker approach in sticklebacks and their potential contribution to discriminate the studied stations.

As observed by Catteau et al. [11], regardless of the station, a high survival rate of fish was noticed (>93.3%), highlighting a good general health status, except for fish caged in *Courrières*, which were characterised by high mortality (63%). No significant weight loss was recorded at all stations (Table S4). These observations allow the interpretation of the physiological responses measured by the multi-biomarker approach after 21 days of caging.

For erythrocyte necrosis, fish from *Etaing* were the most affected. *Etaing* was also characterised by the highest CV for chromosomal damages (3.87 ± 4.72) and had one of the highest lipid peroxidation levels (TBARS, biomarker of cell integrity), higher than reference values established by Catteau et al. [65]. Fish erythrocyte necrosis was most affected at *St Rémy du Nord*, *Courrières* and *Etaing*, with higher responses in males compared to females. Even if biotic or abiotic factors, including gender, influence the responses of some biomarkers, such as AChE, catalase, EROD, GST and immunomarkers [65,66,67,68,69], no study in the literature seems to have highlighted the relationship between erythrocyte necrosis and gender in fish. This latter relationship was not observed for nuclear DNA content or DNA strand breaks in this work. However, considering that gender is essential to analyse erythrocyte necrosis inside the multi-biomarker approach, future investigations could lead to a better understanding of the links between erythrocyte necrosis and gender. In addition, *Courrières* and *Etaing* had the two lowest densities of erythrocytes, which could be correlated with high erythrocyte necrosis. The lifespan of circulating erythrocytes varies in fish between 100 to 500 days [70,71], with a maturation period of 17–23 days [72]. Erythropoiesis is slower than erythrocyte breakdown, which could be impacted by many factors, including environmental toxicants [73]. So, after 21 days of caging, the decline of erythrocytes could be observed before the renewal of blood cells and could be used as an attractive biomarker.

Concerning chromosomal damage, *St Rémy du Nord*, *Artres*, *Biache-St-Vaast* and *Etaing* were characterized by CVs greater than the CVs obtained in reference conditions (−0.96 ± 0.62) (current work, personal communication). The highest CV obtained by FCM was for *Etaing* (3.87 ± 4.72), which could be explained by the increase of the variation in DNA content [74], which resulted from chromosomal aberrations (clastogenic effect) [40] or abnormal cell division (aneugenicity) [75,76]. The CVs obtained for *Courrières* (−0.60 ± 2.18) were the nearest to reference conditions. This station seemed to be less affected by chromosomal damage, but these results came only from the most resistant fish that had survived. One reason for the massive fish mortality recorded in *Courrières* could be related to heavy genomic abnormality with aneuploidy patterns affecting the cytogenetic quality of cells shown in another species in a different context, the blue mussel (*Mytilus* spp.), which hardly withstood the environmental conditions [77]. This high fish mortality can be linked to the high erythrocyte mortality and low erythrocyte density (1.56 ± 0.67 cell/mm^3^ × 10^5^) measured in *Courrières*. *Bouchain* was characterized by a CV (−2.34 ± 1.35) under the CV obtained in reference conditions. So, for *Bouchain*, also, the nuclear DNA content variation is high and could be caused by chromosomal loss or nuclear condensation and shrinkage of fish erythrocytes, marking the early process of cellular mortality [78,79].

In this study, all stations had high levels of DNA strand breaks (between 18.94 ± 7.85% TI and 38.78 ± 19.69% TI) which were two to four times above our reference values. The latter is between reference values measured by Santos et al. and Le Guernic et al. using the same cellular model in sticklebacks [18,35]. Fish caged in *Artres* were the most affected by DNA strand breaks (38.78 ± 19.69% TI) followed by *St Rémy du Nord*, *Biache-St-Vaast*, *Courrières*, *Etaing* and *Bouchain*. Chromosomal aberrations are considered irreversible damages, unlike DNA strand breaks [24,25], but erythrocytes’ DNA-repair capacities in fish species, and specifically in the three-spined stickleback, are not well known. As no decrease in DNA strand breaks was observed after 20 days of depuration following an exposure of 12 days to a well-known genotoxicant, MMS (methylmethanesulfonate, with 0.5 µM and 5 µM tested), Santos et al. supposed that DNA repair activity in stickleback erythrocytes was low [51]. DNA damage would correspond to the accumulation of primary DNA damage until the renewal of erythrocytes. So, although this biomarker does not differentiate stations in this work, if the erythrocyte lifespan is taken into account, the measurement of primary DNA damage in stickleback erythrocytes could be considered an integrative biomarker of genotoxicity [51]. So, the high DNA strand breaks measured in this work could be translated into an environmental degradation that could be linked to a genotoxic effect.

To investigate in more detail the health of sticklebacks caged in each station at the sub-individual level, biomarker responses were integrated into a multivariate analysis (PCA) followed by hierarchical agglomerative clustering (HAC) with the inclusion or not of erythrocyte necrosis and genotoxicity biomarkers. Gathering biomarker responses into multivariate statistics is advised to take into account all biomarker data and improve their interpretation [80]. The inclusion of genotoxic biomarkers inside the classic multi-biomarker approach maintained and affirmed the global separation of the stations. So, both multivariate analyses mainly discriminated two groups of stations, one formed by *Courrières* and *Bouchain* and the second by *St Rémy du Nord*, *Artres*, *Biache-St-Vaast* and *Etaing*. The analysis demonstrated in the first group that fish from *Bouchain* had a similar pattern of biomarkers compared to *Courrières*, being represented by 62.1% and 100% of fish classified in Cluster 1, despite the high fish mortality observed in *Courrières*. Cluster 1, characterised by genotoxicity in terms of negative CVs for DNA content, has allowed us to separate these two stations from the others. In opposition, the new Cluster 5 grouped together half of the fish from *Etaing*, a quarter from *St Rémy du Nord* and some from *Artres* and *Biache-St-Vaast*, which was characterised by a positive CV of the DNA content and by higher erythrocyte necrosis. Biomarkers of genotoxicity and erythrocyte mortality refined the physiological description of the analysis by the formation of a fifth cluster, only built by nuclear DNA content variation and erythrocyte necrosis, improving discrimination among stations. The high DNA strand break level detected in all stations explained the weak discriminating capacity of this biomarker in this study.

Globally, along the Artois-Picardie watershed, fish caged in *Courrières* and *Bouchain* were mainly defined by innate immune responses (high leucocyte respiration, low granulocyte percentages and low phagocytosis efficiency), high cholinesterase activity and negative CVs of nuclear DNA content; *St-Rémy du Nord*, *Artres* and *Biache-St-Vaast* by biomarkers of the antioxidant system (GPx and catalase activities), leucocyte mortality and genotoxicity (increase of CVs, DNA strand breaks) and erythrocyte necrosis; and fish from *Etaing* were characterized by the same biomarkers, apart from leucocyte mortality, and the lowest cholinesterase activity. These biomarkers measured at sub-individual levels translated into a degradation of the general health of the sticklebacks in each station, which can be linked to different environmental stresses along the Artois-Picardie watershed. Land use and the pollution of rivers could explain this degradation (presented in detail in Table S5). For instance, all stations were chemically downgraded for a contamination of benzo(a)pyrene and fluoranthene belonging to the polycyclic aromatic hydrocarbon (PAH) family. In addition, four phytosanitary substances (aclonifene, cypermethrine, chlorpyrifos, bifenox) were measured, which are classified as priorities by the WFD. Notably, some of these molecules are potentially genotoxic substances, according to the literature [81,82,83,84,85,86,87,88]. *Courrières*, with high fish mortality, seemed to be the most degraded. This station is subject to all the contamination coming from upstream because *Courrières* is located at the confluence of two canals (the Deule canal and the Lens canal). High concentrations of organic, trophic and toxic pollutants, which are high and variable in this station, could explain this mortality. In the case of stations showing inconsistency between their chemical status and their ecological parameters under the WFD, as with the six studied stations of the Artois-Picardie watershed, this work has highlighted the relevance of the multi-biomarker approach with the integration of genotoxicity as a complementary indicator to environmental managers in the analysis of the global quality of continental waters.

## 5. Conclusions

This work has expanded the existing battery of biomarkers measured in three-spined sticklebacks during an active biomonitoring of genotoxicity, from DNA strand breaks and chromosomal aberration to erythrocyte mortality. The integration of nuclear DNA content variation and erythrocyte necrosis confirmed and improved the discrimination of the different stations, whereas high DNA strand break levels were detected by the comet assay in all stations. These biomarkers measured at sub-individual levels translated into a degradation of the general health of the fish in each station which can be linked to various environmental stresses along the Artois-Picardie watershed. The most degraded station was *Courrières*, based on the high fish mortality. The multi-biomarker approach with the integration of genotoxicity has allowed us to consider a set of biological effects highlighting the relevance of this approach as a complementary indicator of chemical and ecological parameters of the WFD. These first conclusions were based on studies of six stations along the Artois Picardie watershed. Further research will be carried out on the same stations that will invalidate or confirm these first results. Work is in progress on the three-spined stickleback to define basal values of these biomarkers of genotoxicity and erythrocyte mortality to improve the interpretation of these physiological responses and their reliability in aquatic biomonitoring.

## Figures and Tables

**Table 1:** Characteristics of sites located on the Artois-Picardie watershed: physicochemical parameters measured are expressed as means ± standard deviation; ecological and chemical statuses were collected from the Water Agency of Artois-Picardie databases and are defined by the Water Agency under the evaluation criteria of the Water Framework Directive (WFD, 2000/60/EC).

	Cligneux	Rhonelle	Scarpe	Deule	Sensée	
	St Rémy du Nord	Artres	Biache-Saint-Vaast	Courrières	Bouchain	Etaing
GPS coordinates	N 50°14′20.7″ E 3°53′39.0″	N 50°17′50.4″ E 3°32′40.2″	N 50°18′17.3″ E 2°56′38.1″	N 50°28′08.2″ E 2°56′43.0″	N 50°16′45.4″ E 3°18′18.6″	N 50°16′43.3″ E 2°59′47.8″
Sandre code	01001452	01029000	01037000	01078000	01024000	01000274
WFD data	Yes	Yes	Yes	Yes	Yes	No
Ecological status (2016–2018)	Bad	Mediocre	Mediocre	Mediocre	Mediocre	/
Chemical status (2016–2018)	Bad	Bad	Bad	Bad	Bad	/
Pressure	Poorly urbanised Agricultural practices	Poorly urbanised Agricultural practices	Polluted site Artificial canal	Modified river Waterway transport Urban	Natural stream Down stream	Agricultural practices Up stream
Temperatures (°C)	8.5 ± 2.1	7.0 ± 1.6	10.6 ± 1.5	10.6 ± 2.8	10.2 ± 1.8	10.7 ± 0.9
pH	8.3 ± 0.1	8.3 ± 0.0	8.0 ± 0.1	8.1 ± 0.2	8.0 ± 0.1	7.8 ± 0.0
Conductivity (µS/cm)	625.0 ± 5.6	749.5 ± 4.9	694.5 ± 7.8	719.0 ± 25.5	582.5 ± 47.4	710.0 ± 1.4
Oxygen rate (mg/L)	10.7 ± 0.3	10.8	8.7 ± 1.5	7.5 ± 2.3	6.3 ± 0.9	9.4 ± 0.1

**Table 2:** Biomarker responses assessed in sticklebacks after 21 day of caging in the six sites studied. Data are expressed as means ± standard deviation and letters represent differences between sites (*p* ≤ 0.05) according to a Tukey test or Nemenyi test. n: number of fish dissected for each site; Che: Cholinesterase; EROD: 7-ethoxyresorufin-Odeethylase; GST: glutathione-S-transferase; GSH: total glutathione; GPx: glutathione peroxidase; SOD: superoxide dismutase; CAT: catalase; TBARS: lipid peroxidation; DNA: desoxyribonucleic acid.

			Cligneux	Rhonelle	Scarpe	Deule	Sensée	
			St Rémy du Nord	Artres	Biache-Saint-Vaast	Courrières	Bouchain	Etaing
Biometric index	n	M	14	15	16	5	12	17
		F	14	13	12	6	17	12
	Standard length (mm)	M/F	49.68 ^c^ ±3.53	49.43 ^bc^ ±3.63	46.21 ^a^ ±4.60	44.36 ^a^ ±3.32	46.38 ^ab^ ±3.76	46.21 ^a^ ±3.77
	Weight (g)	M/F	1.87 ^c^ ±0.60	1.62 ^bc^ ±0.34	1.37 ^a^ ±0.42	1.15 ^a^ ±0.22	1.41 ^a^ ±0.61	1.40 ^ab^ ±0.36
	Fulton’s condition	M	1.6 ^a^ ±0.29	1.35 ^a^ ±0.13	1.38 ^a^ ±0.15	1.36 ^a^ ±0.15	1.30 ^a^ ±0.07	1.44 ^a^ ±0.14
		F	1.40 ±0.12	1.29 ±0.10	1.33 ±0.14	1.27 ±0.10	1.51 ±1.03	1.34 ±0.10
	Hepatosomatic index	M	3.55 ±0.67	3.66 ±0.81	3.38 ±0.98	3.15 ±0.53	3.96 ±0.83	3.30 ±1.00
		F	4.27 ±1.08	5.10 ±1.43	4.41 ±1.26	4.08 ±1.16	3.62 ±1.15	3.89 ±0.79
Reproductive system	Gonadosomatic index	M	0.88 ^a^ ±0.31	1.09 ^ab^ ±0.24	1.15 ^b^ ±0.30	1.03 ^ab^ ±0.08	0.98 ^ab^ ±0.16	0.99 ^ab^ ±0.27
		F	2.36 ±0.54	2.08 ±0.54	2.09 ±0.21	2.21 ±0.45	2.04 ±0.54	2.21 ±0.62
Innate immune response	Leucocyte necrosis (%)	M/F	10.46 ^bc^ ±3.77	12.33 ^c^ ±3.54	10.29 ^bc^ ±4.24	6.34 ^a^ ±1.23	8.71 ^ab^ ±4.24	6.99 ^b^ ±2.32
	Leucocyte apoptosis (%)	M/F	8.70 ^c^ ±3.75	7.14 ^bc^ ±3.30	5.27 ^ab^ ±2.53	3.74 ^a^ ±0.99	4.74 ^a^ ±6.31	4.51 ^a^ ±1.84
	Granulocytes–macrophages (%)	M/F	53.47 ^c^ ±7.46	47.84 ^bc^ ±8.06	45.62 ^ab^ ±7.29	40.36 ^ab^ ±7.42	39.52 ^a^ ±9.39	48.30 ^bc^ ±8.77
	Phagocytosis capacity (%)	M	61.11 ^b^ ±6.12	54.80 ^a^ ±4.42	62.15 ^b^ ±4.52	49.01 ^a^ ±3.05	53.68 ^a^ ±4.52	60.11 ^b^ ±5.53
		F	54.96 ^ab^ ±5.56	57.11 ^ab^ ±9.90	63.16 ^c^ ±6.15	51.66 ^a^ ±2.77	51.53 ^a^ ±5.29	56.79 ^bc^ ±2.54
	Phagocytosis efficiency (%)	M/F	16.55 ^b^ ±4.61	19.08 ^b^ ±6.81	25.76 ^c^ ±6.70	10.95 ^a^ ±2.22	13.01 ^a^ ±4.09	18.65 ^b^ ±7.26
	Lysosomal presence (%)	M	173.17 ^a^ ±31.48	234.04 ^ab^ ±38.86	338.26 ^d^ ±73.92	334.09 ^cde^ ±52.36	409.31 ^e^ ±90.56	268.97 ^bc^ ±69.05
		F	214.56 ^a^ ±39.17	227.16 ^bc^ ±44.74	405.29 ^c^ ±149.55	378.98 ^c^ ±66.64	462.36 ^c^ ±78.41	279.43 ^b^ ±77.37
	Respiratory burst index (U)	M/F	1.05 ^bc^ ±0.53	1.15 ^c^ ±0.38	0.93 ^ab^ ±0.30	6.95 ^d^ ±2.38	3.87 ^d^ ±1.94	0.88 ^a^ ±0.50
Neurotoxicity	Che activity (U/g prot)	M/F	38.61 ^b^ ±16.27	37.08 ^b^ ±16.67	27.23 ^a^ ±13.23	65.34 ^c^ ±24.12	43.03 ^b^ ±21.66	25.25 ^a^ ±14.46
Metabolic detoxification	EROD activity (pmol/min/mg prot)	M	5.35 ^bc^ ±4.28	6.11 ^c^ ±3.59	2.73 ^b^ ±1.77	6.56 ^c^ ±1.49	5.80 ^c^ ±3.22	1.11 ^a^ ±0.60
		F	4.04 ^c^ ±1.68	4.38 ^c^ ±1.91	1.85 ^ab^ ±1.19	4.84 ^bc^ ±3.48	4.34 ^c^ ±3.11	1.15 ^a^ ±0.82
	GST activity (U/g prot)	M/F	5083 ±1768	3915 ±1045	4466 ±1710	4038 ±1606	3813 ±1002	4113 ±1085
Antioxidant system	GSH content (µmol/g prot)	M	7.79 ^b^ ±3.57	5.26 ^ab^ ±2.47	6.58 ^ab^ ±3.46	2.44 ^a^ ±3.19	5.55 ^ab^ ±2.68	7.13 ^b^ ±2.80
		F	8.04 ^c^ ±5.80	3.12 ^ab^ ±2.41	6.04 ^bc^ ±3.11	1.60 ^a^ ±1.71	5.19 ^bc^ ±2.37	4.60 ^abc^ ±2.86
	GPx activity (U/g prot)	M	17.49 ±4.76	14.74 ±5.21	17.63 ±11.01	12.53 ±6.64	18.57 ±4.66	16.14 ±5.52
		F	50.22 ^a^ ±25.77	32.49 ^a^ ±15.15	50.58 ^a^ ±21.29	29.55 ^a^ ±13.58	41.23 ^a^ ±17.00	34.57 ^a^ ±16.66
	SOD activity (U/g prot × 10^3^)	M/F	3.14 ±0.78	3.06 ±0.66	2.82 ±1.09	3.06 ±1.13	3.01 ±1.06	3.42 ±0.56
	Catalase activity (U/g prot × 10^5^)	M	4.59 ^b^ ±2.42	2.99 ^ab^ ±1.81	2.01 ^a^ ±1.70	2.27 ^ab^ ±1.52	3.08 ^ab^ ±1.84	4.97 ^b^ ±2.47
		F	7.84 ^ab^ ±2.52	7.97 ^ab^ ±4.40	5.46 ^ab^ ±2.25	3.57 ^a^ ±2.22	6.32 ^ab^ ±2.32	8.94 ^b^ ±4.79
Cell integrity	TBARS (nmol/g prot)	M	131.87 ±37.25	130.14 ±32.40	153.09 ±33.30	142.59 ±27.82	122.70 ±29.66	153.37 ±56.04
		F	82.87 ^a^ ±29.87	123.58 ^ab^ ±48.95	143.19 ^b^ ±23.78	134.90 ^ab^ ±55.11	118.51 ^ab^ ±48.65	143.49 ^b^ ±37.68
Erythrocyte density	Fresh erythrocyte (cell/mm^3^ × 10^5^)	M/F	2.89 ^bc^ ±1.14	3.21 ^c^ ±0.88	2.42 ^b^ ±0.47	1.56 ^a^ ±0.67	2.01 ^a^ ±0.23	1.99 ^a^ ±0.79
Erythrocyte mortality	Erythrocyte necrosis (%)	M	9.21 ^bc^ ±7.50	2.08 ^a^ ±2.28	1.65 ^a^ ±0.75	7.19 ^bc^ ±7.17	2.66 ^ab^ ±1.39	11.99 ^c^ ±7.09
		F	1.56 ^a^ ±0.67	1.34 ^a^ ±0.57	1.39 ^a^ ±0.80	2.13 ^a^ ±1.25	2.10 ^a^ ±0.69	6.11 ^b^ ±3.58
Genotoxicity	DNA strand breaks (tail intensity %)	M/F	28.74 ^ab^ ±16.92	38.78 ^b^ ±19.69	25.28 ^ab^ ±13.69	22.19 ^ab^ ±10.53	18.94 ^a^ ±7.85	22.36 ^a^ ±12.70
	Chromosomal damage	M/F	2.41 ^b^ ±2.35	2.41 ^b^ ±2.19	2.31 ^b^ ±2.78	−0.60 ^a^ ±2.18	−2.34 ^a^ ±1.35	3.87 ^b^ ±4.72

**Table 3:** Composition of the sites using biometric, biochemical and immune biomarkers alone and with the inclusion of genotoxicity. Results are presented as percentages of fish classified in each cluster for each site.

		Cligneux	Rhonelle	Scarpe	Deule	Sensée	
		St Rémy du Nord	Artres	Biache-St-Vaast	Courrières	Bouchain	Etaing
Biometric, biochemical and immune biomarkers	Cluster 1	-	-	-	100	58.6	-
	Cluster 2	-	7.1	67.8	-	13.8	44.8
	Cluster 3	53.6	57.1	17.8	-	20.7	37.9
	Cluster 4	46.4	37.7	14.3	-	6.9	17.2
Genotoxic, biometric, biochemical and immune biomarkers	Cluster 1	-	-	-	100	62.1	-
	Cluster 2	7.1	25	32.1	-	24.1	31.0
	Cluster 3	21.4	39.3	35.7	-	3.5	-
	Cluster 4	46.4	32.1	28.6	-	10.3	17.2
	Cluster 5	25	3.6	3.6	-	-	51.7

**Table 4:** The contribution of current biomarkers with or without integration of genotoxic biomarkers to the building of each cluster in the HAC. The results indicate only the significant v-test obtained for each biomarker and each cluster (│v-test│ ≥ 2.03, *p*-value ≤ 0.05). The most substantially contributing v-tests are represented in bold (│v-test│ ≥ 5.0, *p*-value ≤ 0.05). HSI: hepatosomatic index; GSI: gonadosomatic index; Che: cholinesterase; EROD: 7-ethoxyresorufin-O-deethylase; GST: glutathione-S-transferase; GSH: total glutathione; GPx: glutathione peroxidase; CAT: catalase; SOD: superoxide dismutase; TBARS: thiobarbituric acid reactive substances; DNA: desoxyribonucleic acid.

		Biometric, Biochemical and Immune Biomarkers				Genotoxic, Biometric, Biochemical and Immune Biomarkers				
		Cluster 1	Cluster 2	Cluster 3	Cluster 4	Cluster 1	Cluster 2	Cluster 3	Cluster 4	Cluster 5
Biometric index	HSI	-	−3.2		**5.7**	−2.0	-	−2.5	**5.6**	−3.5
Reproductive system	GSI	-		**−6.4**	**6.1**	-	-	−3.1	**6.5**	−3.9
Innate immune responses	Leucocyte mortality	−3.9	−3.4	3.7	2.9	−4.1	-	**5.7**	-	−2.2
	Granulocyte–macrophage	**−5.6**	-	**5.3**	-	**−5.5**	-	3.8	-	2.1
	Phagocytosis efficiency	**−5.3**	**5.8**	-	-	**−5.1**	2.7	2.6	-	-
	Lysosomal presence	4.7	3.9	−4.8	−3.0	**5.4**	-	-	-	−2.7
	Respiratory burst index	**9.9**	−3.2	−2.9	−2.6	**9.8**	-	−2.1	−2.8	−2.8
Neurotoxicity	Che activity	**5.8**	−2.3	-	-	**5.9**	−3.7	-	-	-
Metabolic detoxification	EROD activity	2.4	−4.9	2.3	-	2.1	-	2.0	-	−2.3
	GST activity	-	−2.8	-	3.7	-	−2.8	-	3.8	-
Antioxidant system	GSH content	−3.4	-	-	-	−3.6	-	-	-	3.4
	GPx activity	-	−2.2	−4.7	**6.8**	-	−2.2	−2.8	**7.8**	−3.1
	SOD activity	-	**5.1**	2.5	-	-	**5.1**	−4.1	-	-
	CAT activity	-	−2.9	−4.4	**7.2**	-	−2.9	−2.4	**7.2**	-
Cell integrity	TBARS content	-	**5.8**	-	−4.1	-	−2.6	2.2	−3.2	4.1
Erythrocyte mortality	Erythrocyte necrosis	-	-	-	-	-	-	-	−2.7	**8.6**
Genotoxicity	DNA strand breaks	/	/	/	/	−2.7	−3.6	4.6	-	-
	Chromosomal damage	/	/	/	/	**−5.9**	-	-	-	**5.4**

## Data Availability

Publicly available datasets were analyzed in this study. This data can be found here: [https://www.eau-artois-picardie.fr/donnees-sur-leau/visualiser-et-telecharger-les-donnees], accessed on 10 January 2022.

## Supplementary Materials

The following supporting information can be downloaded at: https://www.mdpi.com/article/10.3390/toxics10030101/s1: Table S1: Contribution (%) for each biomarker to the construction of the two main components of the PCA taking into account genotoxic and/or biometric, biochemical and innate immune biomarkers (Figure 2 and Figure 3). Variables contributing more than 10% to the building of the axis are indicated in bold. HSI: hepatosomatic index; GSI: gonadosomatic index; Che: cholinesterase; EROD: 7-ethoxyresorufin-Odeethylase; GST: glutathione-S-transferase; GSH: total glutathione; GPx: glutathione peroxidase; SOD: superoxide dismutase; CAT: catalase; TBARS: lipid peroxidation; DNA: deoxyribonucleic acid; Table S2: Results of statistical analysis carried out to assess the differences between stations in terms of sex. Each biomarker was analysed separately via a two-way ANOVA followed by a Tukey test for parametric data or two Kruskal–Wallis tests on site and sex factors separately, followed by a Nemenyi test for non-parametric data (*** = *p* < 0.001, ** = *p* < 0.01, * = *p* < 0.05); Table S3: Biometric index (Fulton’s condition, weight, standard condition) expressed as means ± standard deviation for each station. Stars represent a significant difference obtained between the biometric index at T0 (the first day of the caging) and at T21 (after 21 days of caging in the field) according to a Student’s *t*-test or a Wilcoxon–Mann–Whitney test (* = *p* < 0.05); Table S4: Results of a statistical analysis carried out to assess the difference between each biometric index between T0 and T21 according to a Student’s *t*-test or a Wilcoxon–Mann–Whitney test (* = *p* < 0.05); Table S5: Distribution of land use around each river studied along the Artois-Picardie Watershed in 2018. Downgraded substances were defined according to the criteria of the Water Framework Directive (WFD, 2000/60/EC). Wastewater treatment plants and industries presented correspond to those impacting the water body. (Data of the Artois-Picardie Water Agency (Consulter les données de qualité des rivières|Agence de l’Eau Artois-Picardie (eau-artois-picardie.fr)).
