# Supplementary material for: Integration of Genotoxic Biomarkers in Environmental Biomonitoring Analysis Using a Multi-Biomarker Approach in Three-Spined Stickleback (Gasterosteus aculeatus Linnaeus, 1758)
Source: Toxics. 2022 Feb 22;10(3):101. doi: 10.3390/toxics10030101 (PMC8950626; doi:10.3390/toxics10030101)
Supplement: Supplementary file 1 [file toxics-10-00101-s001.zip › toxics-1572791-supplementary.pdf]

toxics

Supplementary Materials: Integration of Genotoxic Biomarkers in Environmental Biomonitoring Analysis Using a Multi-Biomarker Approach in Three-Spined Stickleback (*Gasterosteus aculeatus* Linnaeus, 1758)

Amélie Cant, Marc Bonnard, Jean-Marc Porcher, Jean Prygiel, Audrey Catteau, Laurence Delahaut, Olivier Palluel, Cyril Turiès, Alain Geffard and Anne Bado-Nilles

Table S1. Contribution (%) for each biomarker to the construction of the two main components of the PCA taking into account genotoxic and/or biometric, biochemical and innate immune biomarkers (Figures 2 and 3). Variables contributing more than 10% to the building of the axis are indicated in bold. HSI: hepatosomatic index; GSI: gonadosomatic index; Che: cholinesterase; EROD: 7-ethoxyresorufin-Odeethylase; GST: glutathione-S-transferase; GSH: total glutathione; GPx: glutathione peroxidase; SOD: superoxide dismutase; CAT: catalase; TBARS: lipid peroxidation; DNA: deoxyribonucleic acid.

		**Biometric, biochemical and immune biomarkers**		**Genotoxic, biometric, biochemical and immune biomarkers**	
		Dim.1 (18.2%)	Dim.2 (15.0%)	Dim.1 (16.8%)	Dim.2 (14.0%)
Biometric index	HSI	10.54	4.10	3.49	12.72
Reproductive system	GSI	0.89	18.54	0.18	16.47
Innate immune responses	Leucocyte mortality	10.29	0.73	6.90	1.36
	Granulocyte-macrophage	9.79	11.28	13.90	1.48
	Phagocytosis efficiency	5.67	6.20	7.80	0.39
	Lysosomal presence	7.35	6.42	11.00	1.22
	Respiratory burst index	14.80	6.80	18.90	0.32
Neurotoxicity	Che activity	6.37	3.37	7.44	0.03
Metabolic detoxification	EROD activity	0.26	3.33	0.18	2.97
	GST activity	6.54	4.68	2.76	4.50
Antioxidant system	GSH content	7.03	0.02	5.67	0.33
	GPx activity	8.20	20.51	0.78	25.67
	SOD activity	1.02	0.46	0.54	0.00
	CAT activity	8.08	12.72	2.24	13.91
Cell integrity	TBARS content	3.17	0.83	0.31	5.99
Erythrocyte mortality	Erythrocyte necrosis	/	/	1.01	9.12
Genotoxicity	DNA strand-breaks	/	/	3.82	0.80
	Chromosomal damage	/	/	13.06	2.70

Table S2. Results of statistical analysis carried out to assess the differences between stations in terms of sex. Each biomarker was analysed separately via a two-way ANOVA followed by a Tukey test for parametric data or two Kruskal–Wallis tests on site and sex factors separately, followed by a Nemenyi test for non-parametric data (*** = $p < 0.001$, ** = $p < 0.01$, * = $p < 0.05$).

p.Value	Site	Sex	Site*Sex	Used Data	Test
Standard lenght	***			Raw	ANOVA
Weight	***		/	Raw	Kruskal-Wallis
K	***	***	/	Raw	Kruskal-Wallis
K - M	**	/	/	Raw	Kruskal-Wallis
K - F		/	/	Raw	Kruskal-Wallis
HSI		***	/	Raw	Kruskal-Wallis
HSI M		/	/	Log-transformed	ANOVA
HSI F		/	/	Raw	Kruskal-Wallis
GSI		***	/	Raw	Kruskal-Wallis
GSI M		/	/	Log-transformed	ANOVA
GSI F		/	/	Raw	Kruskal-Wallis
SPG	***	*	/	Raw	Kruskal-Wallis
SPG M					
SPG F					
L. Necrosis	***		/	Raw	Kruskal-Wallis
L. Apoptosis	***		/	Raw	Kruskal-Wallis
Granulocytes	***			Raw	ANOVA
Phagocytosis capacity	***	*	/	Raw	Kruskal-Wallis
Phago Cap M	***	/	/	Log-transformed	ANOVA
Phago Cap F	***	/	/	Raw	Kruskal-Wallis
Phagocytosis efficiency	***		/	Raw	Kruskal-Wallis
Lysosomal presence	***	**		Log-transformed	ANOVA
Lysosomal presence M	***	/		Raw	ANOVA
Lysosomal presence F	***	/	/	Raw	Kruskal-Wallis
Respiratory burst	***		/	Raw	Kruskal-Wallis
Neurotoxicity	***			Log-transformed	ANOVA
EROD	***	*		Log-transformed	ANOVA
EROD M	***	/	/	Log-transformed	ANOVA
EROD F	***	/	/	Log-transformed	ANOVA
GST			/	Raw	Kruskal-Wallis
GPx		***	/	Raw	Kruskal-Wallis
GPx M		/	/	Raw	Kruskal-Wallis
GPx F	*	/	/	Log-transformed	ANOVA
GSH	***	*	/	Raw	Kruskal-Wallis
GSH M	*	/	/	Raw	ANOVA
GSH F	**	/	/	Raw	Kruskal-Wallis
SOD			/	Raw	Kruskal-Wallis
CAT	**	***	/	Raw	Kruskal-Wallis
CAT M	**	/	/	Raw	Kruskal-Wallis
CAT F	*	/	/		
TBARS	***	*		Raw	ANOVA
TBARS M		/	/	Raw	ANOVA
TBARS F	**	/	/	Raw	ANOVA
Erythrocyte density	***		/	Raw	Kruskal-Wallis
Erythrocytes necrosis	***	**	/	Raw	Kruskal-Wallis
Erythrocytes necrosis M	***	/	/	Raw	Kruskal-Wallis

Erythrocytes necrosis F	***	/	/	Log-transformed	ANOVA
DNA strand breaks	**		/	Raw	Kruskal-Wallis
Chromosomal damages	***		/	Raw	Kruskal-Wallis

Table S3. Biometric index (Fulton's condition, weight, standard condition) expressed as means ± standard deviation for each station. Stars represent a significant difference obtained between the biometric index at T0 (the first day of the caging) and at T21 (after 21 days of caging in the field) according to a Student's *t*-test or a Wilcoxon–Mann–Whitney test (* = $p < 0.05$).

	**Standard length T0 (mm)**	**Standard length T21 (mm)**	**Weight T0 (g)**	**Weight T21 (g)**	**Fulton's condition index T0**	**Fulton's condition index T21**
	Mean ± SD	Mean ± SD	Mean ± SD	Mean ± SD	Mean ± SD	Mean ± SD
St Rémy du Nord	49.03 ± 4.13	49.68 ± 3.53	1.60 ± 0.41	1.87 ± 0.6 *	1.35 ± 0.19	1.50 ± 0.24 *
Artres	49.03 ± 3.59	49.43 ± 3.63	1.66 ± 0.37	1.61 ± 0.34	1.39 ± 0.09	1.32 ± 0.12 *
Biache-Saint-Vaast	46.80 ± 5.22	46.21 ± 4.60	1.43 ± 0.45	1.37 ± 0.42	1.37 ± 0.19	1.36 ± 0.14
Courrières	45.67 ± 4.29	44.63 ± 3.32	1.30 ± 0.34	1.15 ± 0.22	1.35 ± 0.15	1.31 ± 0.13
Bouchain	46.17 ± 3.86	46.38 ± 3.76	1.36 ± 0.36	1.41 ± 0.61	1.36 ± 0.12	1.29 ± 0.10 *
Etaing	47.07 ± 3.94	46.21 ± 3.77	1.45 ± 0.36	1.4 ± 0.36	1.37 ± 0.14	1.40 ± 0.13

Table S4. Results of a statistical analysis carried out to assess the difference between each biometric index between T0 and T21 according to a Student's *t*-test or a Wilcoxon–Mann–Whitney test (* = $p < 0.05$).

**K**	**Sites**	***p*.values ("two.sided")**	***p*.values ("less or greater")**	**Difference**	**Used data**	**Test (Independent data)**
	St Rémy du Nord	*p*.values = 0.02394	*p*-value = 0.01197	KT0 < KT21 *	Raw	Wilcoxon
	Artres	*p*-value = 0.01527	*p*-value = 0.007634	KT0 > KT21 *	Log-transformed	Student
	Biache-Saint-Vaast	*p*.values = 0.9441	/		Raw	Wilcoxon
	Courrières	*p*.values = 0.6584	/		Raw	Wilcoxon
	Bouchain	*p*-value = 0.01289	*p*-value = 0.006446	KT0 > KT21 *	Raw	Student
	Etaing	*p*-value = 0.4174	/		Raw	Wilcoxon

**Weight**	**Sites**	***p*.values ("two.sided")**	***p*.values ("less or greater")**	**Difference**	**Used data**	**Test (Independent data)**
	St Rémy du Nord	*p*-value = 0.02168	*p*-value = 0.01084	WeT0 < WET21 *	Raw	Wilcoxon
	Artres	*p*-value = 0.621	/		Raw	Student
	Biache-Saint-Vaast	*p*-value = 0.5853	/		Raw	Student
	Courrières	*p*-value = 0.1853	/		Raw	Student
	Bouchain	*p*-value = 0.5291	/		Log-transformed	Student
	Etaing	*p*-value = 0.1871	/		Raw	Wilcoxon

Standard lenght	Sites	*p*.values ("two.sided")	*p*.values ("less or greater")	Difference	Used data	Test (Independent data)
	St Rémy du Nord	*p*-value = 0.5265	/		Raw	Student
	Artres	*p*-value = 0.6781	/		Raw	Student
	Biache-Saint-Vaast	*p*-value = 0.653	/		Raw	Student
	Courrières	*p*-value = 0.3683	/		Raw	Student
	Bouchain	*p*-value = 0.8268	/		Log-transformed	Student
	Etaing	*p*-value = 0.6781	/		Raw	Student

Table S5. Distribution of land use around each river studied along the Artois-Picardie Watershed in 2018. Downgraded substances were defined according to the criteria of the Water Framework Directive (WFD, 2000/60/EC). Wastewater treatment plants and industries presented correspond to those impacting the water body. (Data of the Artois-Picardie Water Agency (Consulter les données de qualité des rivières | Agence de l'Eau Artois-Picardie (eau-artois-picardie.fr)).

	Cligneux	Rhonelle	Scarpe	Deule	Sensée
Body of surface water	Natural	Natural	Strongly modified	Artificial	Natural
Artificial territory	240 (7.1)	4 483 (30.4)	3802 (25.9)	13003 (45.2)	2795 (16.0)
Forest and natural area	183 (5.4)	272 (1.9)	342 (2.3)	1161 (4.0)	445 (2.5)
Wetland	0 (0.0)	31 (0.2)	201 (1.4)	0 (0.0)	553 (3.2)
Agricultural territory	2970 (87.5)	9885 (67.0)	10243 (69.8)	14589 (50.7)	13487 (77.1)
Wastewater treatment plants	2	5	8	8	5
Industries	5	56	78	143	14
Downgrading substance	PAH	PAH	PAH	PAH and Pb	PAH

Data are expressed in hectare (% of land use distribution). PAH: polycyclic aromatic hydrocarbon. PB: lead.
